# Development of the Diabetes Burnout Scale for patients with type 2 diabetes: a modified Delphi study

**DOI:** 10.3389/fendo.2026.1767244

**Published:** 2026-03-23

**Authors:** Yun Wei, Yiling Niu, Qiumei Cao, Yanhua An, Wei Feng, Zhaolu Pan

**Affiliations:** Department of General Practice, Beijing Tongren Hospital, Capital Medical University, Beijing, China

**Keywords:** Delphi, diabetes burnout, measurement development, scale, type 2 diabetes

## Abstract

**Background:**

Enhancing diabetes outcomes and delivering patient-centered care hinge on a thorough understanding of diabetes-related burnout. This study aimed to develop an evidence-based, consensus-guided scale to assess diabetes burnout in adults with type 2 diabetes (T2D).

**Methods:**

A modified Delphi method was adopted. Key dimensions and potential items were identified through a literature review and qualitative patient interviews. A structured Delphi process was then used to establish expert consensus with a diverse panel comprising endocrinologists, general practitioners, and patient experts with T2D. The expert panel reviewed the draft over two rounds between April and July 2025, with consensus meetings convened after each round. The initial conceptual framework was refined through this iterative process.

**Results:**

Twenty-three experts (median age 48 years [IQR 40, 54]; 87% female) participated in both rounds of the Delphi survey. After the first round, the description of one dimension and 10 items were modified. One item was split into two separate items based on expert recommendations, and two items were deleted because they did not achieve consensus. A new dimension, termed “Psychological stress” was suggested by more than two experts, along with four additional items. After the second round, all dimensions and items achieved consensus in terms of importance and feasibility. The final Diabetes Burnout Scale for T2D (DBS-T2D) comprised 26 items organized into four dimensions: psychological stress, emotional exhaustion, physical fatigue, and disengagement from diabetes management.

**Conclusions:**

The DBS-T2D was systematically developed in this study through a transparent and replicable process. Future work should focus on the scale’s psychometric validation and the development of evidence-based interventions to alleviate burnout and enhance long-term health outcomes.

## Introduction

Diabetes mellitus (DM) represents a significant and increasingly prevalent global health challenge. According to the International Diabetes Federation (IDF), approximately 589 million adults (one in nine) were living with diabetes worldwide in 2024, a number projected to rise to 853 million by 2050. In 2024, diabetes accounted for 3.4 million deaths and incurred a global health expenditure of USD 1.015 trillion (1). More than 90% of individuals with diabetes have type 2 diabetes (T2D). The adverse effects of diabetes can be mitigated through preventive strategies targeting T2D, as well as through early diagnosis and appropriate management for all types of diabetes. Such interventions can assist individuals with diabetes in avoiding or delaying complications (1).

Individuals with diabetes encounter a wide range of complex and multifaceted psychological challenges, including depression, distress, and burnout. Diabetes burnout has been recognized as a significant healthcare concern since the early 1980s (2), with researchers observing that “patients, as well as healthcare professionals, can suffer burnout.” It is conceptualized as a psychological response characterized by emotional exhaustion and disengagement experienced by individuals managing this chronic condition. Diabetes burnout arises from an imbalance between the demands of diabetes management and an individual’s capacity to meet these demands, potentially reflecting a maladaptive coping mechanism and a defense strategy in response to unresolved distress (2, 3). Over the past few years, Abdoli et al. have conducted extensive research on the concept of diabetes burnout (4–9), identifying three interdependent dimensions: exhaustion, detachment, and loss of control. These dimensions interact dynamically, resulting in various diabetes burnout profiles that individuals may experience throughout their journey with the condition (4–6, 9).

Diabetes burnout represents a significant barrier to glycemic control and treatment adherence, potentially leading to neglect or even abandonment of self-care behaviors, thereby increasing the risk of diabetes-related complications (4, 10). In the DAWN study, diabetes burnout—along with depression, anxiety, and stress—was identified as one of the main psychosocial complications of diabetes by 66%–74% of healthcare providers (11). In a study of individuals with T2D, approximately 36% of participants reported experiencing diabetes burnout, which hindered their medication adherence (12).

However, no reliable and valid instrument currently exists that is specifically designed to assist researchers and clinicians in identifying diabetes burnout among individuals with T2D. While validated instruments are available, they primarily focus on assessing diabetes distress (13, 14), depressive symptoms (15), or diabetes burnout specifically in patients with type 1 diabetes (T1D) (16). The Maslach Burnout Inventory, traditionally used to measure occupational burnout, has been applied to chronic illness contexts. However, while managing diabetes can be likened to a full-time job, occupational burnout measures fail to capture the unique characteristics of diabetes as a chronic and demanding condition. Although recent studies (4–9) have identified a conceptual overlap between the powerlessness subscale of the Type 1 Diabetes Distress Scale (T1-DDS) and the loss of control dimension in diabetes burnout—both of which assess the emotional burden of diabetes—existing diabetes distress measures do not adequately capture the distinct dimensions of diabetes burnout. For instance, the Problem Areas in Diabetes Scale (PAID) (14) focuses on emotional distress associated with living with diabetes, while the T1-DDS (13) emphasizes sources of distress specific to adults with T1D. In 2021, the Diabetes Burnout Scale (DBS) was developed for adults with T1D, based on a conceptual framework that evaluates three primary dimensions: exhaustion, detachment, and loss of control (16). The items within each dimension, as well as the total DBS score, reflect the experiences of adults with T1D undergoing diabetes burnout (16).

The management of T1D is akin to a precise science, with its cornerstone being insulin replacement. Burnout in type 1 diabetes is frequently linked to the relentless cognitive burden of self-management and the pervasive fear of acute metabolic decompensation (e.g., severe hypoglycemia or diabetic ketoacidosis), reflecting an exhaustion stemming from the constant threat to physiological stability. In contrast, the management of T2D constitutes a dynamic, progressive, and multifactorial intervention process, including lifestyle modification as the bedrock, adherence to a stepped and individualized pharmacological strategy, the establishment of personalized glycemic targets, and holistic management of cardiovascular and renal risk factors. In type 2 diabetes mellitus, burnout often manifests in patterns of behavioral dysregulation—oscillating between strict adherence and lapses—coupled with guilt (e.g., following dietary indiscretions), intentional omission of medication due to the absence of immediate somatic feedback, and avoidance of medical follow-up driven by fear of perceived criticism from healthcare providers. Enhanced diabetes outcomes and the delivery of patient-centered care will depend on a thorough understanding of diabetes-related burnout, as well as the implementation of appropriate interventions to address existing gaps where necessary. Currently, no validated instrument exists to specifically capture the manifestations of burnout in individuals with type 2 diabetes. To address this gap, this study sought to develop a novel scale—the Diabetes Burnout Scale for patients with T2D (DBS-T2D)—designed to systematically assess this clinically relevant but under-researched phenomenon.

## Methods

### Design

A modified Delphi method was adopted in the study, which was the most widely used method for selecting quality indicators in healthcare (17, 18). The modified Delphi technique was chosen over other consensus methodologies due to its ability to be conducted electronically, its capacity to recruit a diverse range of experts across time and geographical locations, the anonymity it provides during the consensus process, and its effectiveness in avoiding groupthink and undue influence from more vocal members of an expert panel (19).

**Figure 1:**
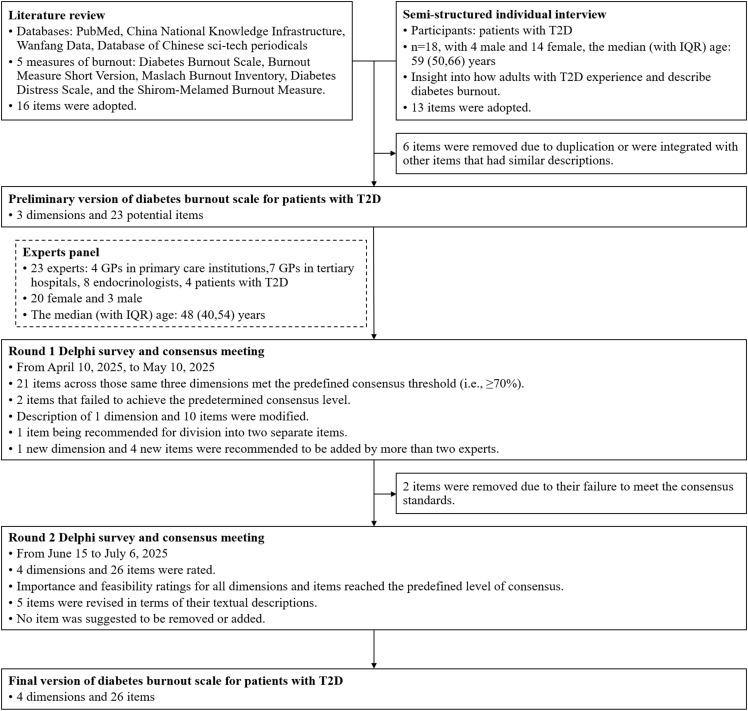
Flow diagram of the process of developing a diabetes burnout scale for parents with type 2 diabetes.

The development of the DBS-T2D consisted of three distinct stages: (1) questionnaire preparation, during which an initial pool of items related to diabetes burnout was generated through a literature review and semi-structured individual interviews with T2D patients; (2) Delphi survey, wherein a two-round modified Delphi method was employed to achieve consensus on the framework structure, terminology, dimensions, and items; (3) consensus meeting, during which the precise wording of the items was finalized (**Figure 1**).

### Patient and public involvement

Four patients with experience of diabetes burnout were actively involved as expert panelists throughout the Delphi process.

### Questionnaire preparation

In this study, diabetes burnout is defined as a state of emotional, physical, and behavioral exhaustion experienced by patients with T2D during the self-management process, resulting from prolonged exposure to diabetes-related stress. Potential items were generated through a literature review and semi-structured individual interviews with T2D patients.

The initial pool of items related to diabetes burnout was derived from existing scales identified through a literature review. A literature search was conducted in PubMed and three Chinese databases (China National Knowledge Infrastructure, Wanfang Data, and the Database of Chinese Sci-Tech Periodicals) using search terms related to diabetes burnout assessment. Through this process, we identified five burnout-related measures: the Diabetes Burnout Scale (16), the Burnout Measure Short Version (20, 21), the Maslach Burnout Inventory (22), the Diabetes Distress Scale (23), and the Shirom-Melamed Burnout Measure (24). Potential items were extracted and screened by two reviewers (Wei Y and Niu YL) according to the following criteria: (a) the items were consistent with the characteristics typically associated with diabetes burnout; (b) the items were applicable to T2D populations; and (c) the items were measurable. A total of 16 items were derived from the literature review.

Then we conducted semi-structured individual interviews to show us with insight into how adults with T2D experience and describe diabetes burnout. Patients aged 18–75 years with a confirmed diagnosis of type 2 diabetes and self-reported experience of diabetes burnout were recruited for semi-structured individual interviews, provided they could communicate coherently. The interviews explored patients’ experiences, manifestations, timing, and influencing factors of diabetes burnout. All interviews were audio-recorded, transcribed verbatim, and independently coded by two researchers (Wei Y and Niu YL). Any discrepancies in coding were resolved through team discussion until consensus was reached. Finally, 18 patients with type 2 diabetes (4 male, 14 female; median age 59 years [IQR 50–66]) participated in the interviews. Illustrative quotes from the identified themes and subthemes were used to inform item generation, resulting in 13 items derived from the semi-structured interview process.

A total of 29 items were identified through the two processes described above. These items were then systematically reviewed during a research team meeting attended by two endocrinologists and three general practitioners. After deleting duplicate items and merging those with similar descriptions, a preliminary list of 23 potential items was developed and categorized into three corresponding dimensions of diabetes burnout: emotional exhaustion (n = 9), physical exhaustion (n = 5), and disengagement from diabetes management (n = 9).

### Delphi survey

#### Expert panel

We employed purposive sampling to recruit a diverse panel of experts, which included endocrinologists, general practitioners (GPs) from both tertiary hospitals and primary care institutions, and patients with T2D. Potential participants were required to meet the following inclusion criteria: (a) at least 10 years of experience in the management or self-management of T2D; (b) for endocrinologists and GPs, holding a senior clinical position; (c) for patients, experiencing diabetes burnout and possessing adequate comprehension and expressive abilities to participate effectively in the study. Individuals with experience in managing type 1 diabetes or gestational diabetes, either through clinical practice or self-management, were excluded from this study. Although there is no consensus in the literature regarding the optimal size of a Delphi expert panel (19, 25, 26), previous studies have suggested that a sample of at least 15 participants is sufficient to provide representative information (27, 28). Ultimately, 26 qualified experts were invited via email, and 23 agreed to participate in this study.

### Delphi process

There is no strict limitation on the number of rounds that can be conducted (29). The number of rounds depends on the amount of time available, whether the researcher has indicated the Delphi sequence with one broad question or with a list of questions, and consideration of levels of sample fatigue. The literature demonstrates that the classic Delphi technique had four rounds (28); however, more recent evidence appears to show that either two or three rounds are preferred (30, 31). The Delphi process is terminated once the items under investigation reach a predefined consensus threshold. There are no universally accepted consensus criteria for Delphi studies (32, 33). To address this, the research team predefined consensus criteria before conducting the Delphi survey. Consensus was determined based on two criteria applied to both importance and feasibility (30): (a) a median score of 7 or higher on a 9-point scale, and (b) at least 70% of panel members rating the item as 7 or higher.

#### First round

The first round of the Delphi survey was conducted over a one-month period, from April 10 to May 10, 2025. Each expert panelist received a first-round questionnaire by WeChat, which included the research background, project objectives, a demographic information form, detailed scoring instructions, and a comprehensive list of potential diabetes burnout dimensions and items, each accompanied by a descriptive explanation. The importance and feasibility of each dimension and item were assessed using a 9-point Likert scale. For example, for the item “1.1 Self-management of diabetes-related stress, ” participants were instructed to rate its importance and feasibility in assessing diabetes burnout on a 9-point Likert scale (1 = not important or feasible at all; 9 = very important or feasible). Panelists were also provided with the opportunity to submit free-text comments on existing dimensions and items. In addition, the questionnaire included designated sections where experts could propose supplementary dimensions and items they believed should be incorporated. Responses from each expert were de-identified with a unique code to protect their identity. All data were kept confidential and used for research purposes only.

Following the first round of the Delphi survey, data were systematically collected and analyzed. The results, including the median scores, frequency distributions of responses, and qualitative comments, were documented and presented. Each dimension and item was reviewed and discussed by the research group based on its rating results and the associated qualitative feedback. Dimensions and items that reached the consensus threshold or were revised based on expert input were retained for inclusion in the second-round questionnaire. Additionally, new dimensions and items were incorporated into the second-round survey if suggested by more than two experts. Conversely, dimensions and items that failed to achieve consensus or were recommended for removal by more than two experts were excluded from subsequent rounds (31, 34, 35).

#### Second round

The second round of the Delphi survey was conducted from June 15 to July 6, 2025, spanning a period of three weeks. The dimensions and items related to diabetes burnout that were provisionally confirmed in the first round were compiled into a structured questionnaire for the second-round survey. This questionnaire was distributed to the same panel of experts by WeChat, accompanied by a visual report summarizing the outcomes of the first round. Each dimension and item was evaluated in terms of importance and feasibility using the same 9-point Likert scale as employed in the first round. In addition, participants were invited to propose new dimensions or items, express agreement or disagreement with the existing ones, and provide feedback on the wording and clarity of the content.

### Consensus meeting

A face-to-face consensus meeting was convened after each round of the Delphi process. The meeting was attended by a subgroup of the 23 Delphi panelists (n = 7) who were able to participate in person, consisting of 2 endocrinologists, 3 general practitioners, and 2 patients with type 2 diabetes. A comprehensive document summarizing the scoring results and qualitative feedback was presented to the panel for discussion. Following the presentation, panel members engaged in focused deliberations on the feasibility of including dimensions and items, as well as their respective descriptions and wording. Items that received a marginal level of agreement (i.e., between 60.0% and 69.9%) were specifically highlighted and reviewed. Consensus was ultimately achieved through structured discussion.

### Statistical analysis

Two researchers (Wei Y and Niu YL) concurrently established the database and entered data using Epidata 3.0. Any discrepancies or errors were resolved by a third researcher through verification and correction. Descriptive analysis was conducted to summarize the characteristics of participants and study outcomes. Continuous variables were presented as medians with interquartile ranges (IQR), whereas categorical variables were described using frequencies and percentages. The rating results for each dimension and item were reported in terms of median scores and score distributions. Data management and statistical analysis were performed using SPSS version 22.0. All qualitative feedback provided by experts was systematically extracted and categorized, including suggestions for revising, deleting, or adding dimensions and items. The frequency of identical or similar suggestions was also documented. Data from Round 1 were analyzed in May 2025, and data from Round 2 were analyzed in July 2025.

## Results

### Panel characteristics in Delphi survey

All 23 experts participated in both rounds of the Delphi survey, of whom 20 were female (87.0%) and 3 were male (13.0%). The median age of the experts was 48 years (interquartile range: 40-54). Among them, 19 experts were based in Beijing, while the remaining 4 were from four provinces across China: Hainan, Shandong, Ningxia, and Henan. Endocrinologists constituted 34.8% of the panel, GPs working in primary care institutions accounted for 17.4%, GPs in tertiary hospitals made up 30.4%, and individuals with T2D comprised the remaining 17.4% (n=4). A total of 82.6% of the experts held a master’s or doctoral degree, and 78.3% held senior professional titles. Professional titles were not applicable to the four participants with T2D. Additionally, 87.0% of the experts had more than 10 years of experience in diabetes management and self-management education. The characteristics of participants in the Delphi survey are reported in **Table 1**.

**Table 1:** Panel characteristics of the Delphi process (n=23).

Characteristics	Frequency	Percentage (%)
Gender		
Male	3	13.0
Female	20	87.0
Age, years		
30-39	4	17.4
40-49	10	43.5
≥ 50	9	39.1
Professional field		
GPs in primary care institutions	4	17.4
GPs in tertiary hospitals	7	30.4
Endocrinologists	8	34.8
Patients with T2D	4	17.4
Years of experience in diabetes management and self-Management		
< 10	3	13.0
10-19	10	43.5
20-29	7	30.4
≥ 30	3	13.0
Highest degree		
PhD	9	39.1
Master	10	43.5
Bachelor	3	13.0
Junior college degree	1	4.3
Professional title*		
Middle grade title	1	4.3
Associate senior grade title	6	26.1
Senior grade title	12	52.2
Not applicable	4	17.4

GP, general practitioner; PhD, doctor of philosophy; T2D, type 2 diabetes.

*In China, the professional titles for physicians are categorized into four distinct levels: junior grade (resident physician), intermediate grade (attending physician), deputy senior grade (deputy chief physician), and senior grade (chief physician). These classifications are determined by the healthcare professionals’ work experience and research accomplishments. This question in the questionnaire is not applicable to patients with type 2 diabetes.

### Delphi survey and consensus meeting

As shown in **Table 2**, during the first round, the median scores for importance and feasibility across all three dimensions and 23 items ranged from 7.00 to 9.00. Importance and feasibility ratings for all three dimensions achieved consensus, with the percentage of panel ratings falling within the top tertile (7–9) ranging from 73.9% to 95.7%. Consensus was also reached for the importance and feasibility of all items, except for item Diabetes management can be physically challenging for patients and item Diabetes management requires patients to work hard, which did not meet the 70.0% agreement threshold for feasibility [for both items, 69.6% of experts rated in the top tertile (7–9)].

**Table 2:** Results of the Delphi process.

Domains and items	Round 1				Round 2			
	Importance		Feasibility		Importance		Feasibility	
	Median	Agreement (7-9)	Median	Agreement (7-9)	Median	Agreement (7-9)	Median	Agreement (7-9)
1.Psychological stress*					9	95.7%	8	87%
1.1 Self-management of diabetes-related stress	9	82.6%	8	91.3%	9	87%	8	91.3%
1.2 Excessive concern about diabetes complications*					8	87%	8	91.3%
1.3 Economic burden*					8	82.6%	8	87%
1.4 Time constraints*					8	82.6%	8	87%
1.5 Social pressure *					8	82.6%	8	91.3%
2.Emotional exhaustion	9	95.7%	8	87%	8	95.7%	8	87%
2.1 Daily diabetes management consumes too much energy from patients	8	78.3%	8	91.3%	9	91.3%	8	91.3%
2.2 Daily diabetes management makes patients feel emotional exhausted	8	82.6%	8	73.9%	8	87%	8	87%
2.3 Daily diabetes management makes patients feel bored	8	87%	8	78.3%	8	95.7%	8	82.6%
2.4 Loss of confidence in diabetes management	8	91.3%	8	87%	8	91.3%	8	87%
2.5 It is believed that diabetes management is meaningless	8	87%	8	82.6%	9	87%	8	91.3%
2.6 Daily diabetes management deprives patients of the joy of life	8	87%	8	87%	9	95.7%	8	95.7%
2.7 A lack of family support makes patients feel isolated	8	82.6%	7	87%	8	82.6%	7	82.6%
2.8 Insufficient medical support makes patients feel helpless					8	82.6%	8	87%
2.9 There is an idea of discontinuing diabetes management	9	91.3%	8	87%	9	87%	8	87%
3.Physical Fatigue	8	82.6%	8	73.9%	8	91.3%	8	87%
▲Diabetes management can be physically challenging for patients	8	78.3%	7	69.6%				
3.1 Diabetes management requires considerable effort from patients	8	91.3%	8	87%	8	91.3%	8	91.3%
▲Diabetes management needs patients work hard	7	78.3%	7	69.6%				
3.2 Daily diabetes management makes patients feel physical exhausted	8	82.6%	8	82.6%	8	91.3%	8	95.7%
3.3 Diabetes management affects patients’ daily lives	8	87%	8	82.6%	8	87%	8	82.6%
4.Disengagement from diabetes management	9	95.7%	9	91.3%	9	100%	8	82.6%
4.1 It is acceptable for patients to neglect diabetes management	9	91.3%	8	87%	9	91.3%	8	87%
4.2 Disengagement from diabetes management makes patients feel relaxed	8	91.3%	8	87%	9	95.7%	8	91.3%
4.3 No longer adhere to diabetes diet management	9	91.3%	8	87%	9	91.3%	8	82.6%
4.4 No longer engage in exercise management for diabetes	9	95.7%	8	95.7%	9	91.3%	9	82.6%
4.5 Failing to take hypoglycemic drugs or inject insulin on time	9	91.3%	8	91.3%	9	91.3%	9	95.7%
4.6 Failing to monitor blood glucose regularly	9	95.7%	8	100%	9	91.3%	8	91.3%
4.7 Whether the glycemic result is within the standard did not cause the patient any concern	9	95.7%	8	95.7%	8	95.7%	8	95.7%
4.8 Poor glycemic results cannot prevent patients from disengaging from diabetes self-management	9	100%	8	95.7%	9	95.7%	8	95.7%
4.9 Poor glycemic results cannot prompt patients to seek medical help	9	95.7%	8	95.7%	9	87%	8	91.3%

Domains and items in the table are modified versions after two rounds of consultation; experts rated the importance and feasibility of each domain and item on a 1–9 Likert scale (1 = not important/feasible and 9 = very important/feasible).

*Items were removed after the first round due to their failure to meet the consensus standards.

▲Items were recommended to be added.

From an initial pool of three dimensions comprising 23 items, 21 items across those same three dimensions met the predefined consensus threshold (i.e., ≥70%) following the first round and were therefore carried forward into the second round (see **Figure 1**). Two items that failed to achieve the predetermined consensus level were removed from the list. A total of 66 free-text suggestions were received, addressing various aspects such as the overall structure of the framework, the wording and description of existing items, the division of certain items, and proposals for new additions. Following qualitative analysis and a consensus meeting, two items were removed due to their failure to meet the consensus standards. One item (1.9: “Diabetes management leaves patients feeling isolated and helpless”) was recommended for division into two separate items. Additionally, one dimension and 10 items underwent rewording, while one new dimension and four new items were recommended for addition. Adjustments to all dimensions and items after the first round of the Delphi survey and consensus meeting are shown in **Table 3**. Consequently, a total of four dimensions and 26 items proceeded to the second round of expert consultation.

**Table 3:** Adjustment during the two rounds of Delphi survey and consensus meeting.

Delphi survey		Domains and items	Adjustment
First round	**Removement**	2.1Diabetes management can be physically challenging for patients	removed due to failure to meet the consensus standards
		2.3Diabetes management needs patients work hard	
	**Addition**	1.Psychological stress	suggested to added by more than two experts
		1.2 Excessive concern about diabetes complications	
		1.3 Economic burden	
		1.4 Time constraints	
		1.5 Interpersonal relationship stress	
	**Modification**	2.Physical exhaustion	3. Physical fatigue
		1.1 Diabetes makes patients feel very stressed	modified to “1.1 Self-management of diabetes-related stress” under the domain “Psychological stress”
		1.3 Diabetes management makes patients feel mentally exhausted	2.2 Daily diabetes management makes patients feel emotional exhausted
		1.7 It is believed that standardized diabetes management has no significance at all	2.5 It is believed that diabetes management is meaningless
		1.9 Diabetes management leaves patients feeling isolated and helpless	divided into item “2.7 A lack of family support makes patients feel isolated” and item “2.8 Insufficient medical support makes patients feel helpless”
		1.10 There is an idea of giving up managing diabetes	2.9 There is an idea of discontinuing diabetes management
		3.2 Failure to follow a diabetic diet	4.3 No longer adhere to diabetes diet management
		3.3 Failure to conduct regular exercise management	4.4 No longer engage in exercise management for diabetes
		3.4 Failure to receive diabetes medication treatment on time	4.5 Failing to take hypoglycemic drugs or inject insulin on time
		3.7 Getting out of diabetes management makes patients feel very happy	4.2 Disengagement from diabetes management makes patients feel relaxed
		3.8 Patients with poorly controlled blood glucose is still disengaged from diabetes management	4.8 Poor glycemic results cannot prevent patients from disengaging from diabetes self-management
		3.9 Patients with poorly controlled blood glucose still refuse to seek medical treatment	4.9 Poor glycemic results cannot prompt patients to seek medical help
Second round	**Removement**	–	–
	**Addition**	–	–
	**Modification**	1.5 Interpersonal relationship stress	1.5 Social pressure
		2.1 Diabetes management takes up too much of patients’ energy every day	2.1 Daily diabetes management consumes too much energy from patients
		2.3 Diabetes management makes patients feel bored	2.3 Daily diabetes management makes patients feel bored
		4.6 Do not monitor blood sugar	4.6 Failing to monitor blood glucose regularly
		4.7 Not care about glycemic results	4.7 Whether the glycemic result is within the standard did not cause patients any concern

In round 2, the median scores for importance and feasibility across all four dimensions and 26 items ranged from 7.00 to 9.00. Importance and feasibility ratings for all dimensions and items reached the predefined level of consensus and were included in the framework, with the percentage of panel ratings falling within the top tertile (7–9) ranging from 82.6% to 100% (see **Table 2**). Thirteen free-text suggestions were received, concentrating on the wording and description of existing items. After a consensus meeting, only five items were revised in terms of their textual descriptions. No item was suggested to be removed or added. Adjustments after the second round of the Delphi survey and consensus meeting are also shown in **Table 3**.

Finally, the DBS-T2D included 26 items, which were organized into four dimensions: psychological stress (5 items), emotional exhaustion (9 items), physical fatigue (3 items), and disengagement from diabetes management (9 items).

## Discussion

To our knowledge, this is the first study to develop a comprehensive set of items through consensus to assess diabetes burnout experienced by individuals with T2D. This study followed a rigorous process that involved a multi-method approach to identify and analyze indicators associated with diabetes burnout, including a literature review, semi-structured individual interviews with patients with T2D, a modified Delphi survey, and a consensus meeting. The Delphi process assembled a panel of experts with diverse professional backgrounds, varied roles in diabetes management, and representation from different geographical regions to ensure broad applicability and relevance to a national audience. The final DBS-T2D consisted of four dimensions and 26 items.

### Comparison with existing instruments

As no formal definition of type 2 diabetes (T2D)-specific burnout currently exists, we developed one for this study. Our definition draws on: (a) burnout constructs from type 1 diabetes research (6–8); (b) the diabetes distress literature (2, 3, 13, 14); and (c) findings of our preliminary semi-structured interviews with T2D patients. Accordingly, diabetes burnout is defined in this study as a state of emotional, physical, and behavioral exhaustion experienced by patients with T2D during the self-management process, resulting from prolonged exposure to diabetes-related stress. It is characterized by feelings of fatigue and frustration toward diabetes management tasks, and may manifest as disengagement from self-care behaviors. Therefore, the three initial evaluation dimensions (emotional exhaustion, physical fatigue, and disengagement from diabetes management) were established based on literature review and patient interviews.

A review of the literature revealed only one instrument, the DBS, specifically designed to assess diabetes burnout in diabetes patients (16), which relies on a conceptualization of burnout in adults with T1D by assessing three main dimensions including exhaustion, detachment, and loss of control (16). Several key evaluation indicators in the DBS-T2D overlap considerably with those in the DBS, such as “emotionally exhausted, ” “physically exhausted, ” “neglecting diabetes management, ” and indicators related to diabetes self-management failure behaviors. Our findings also suggest, based on the semi-structured individual interviews, that diabetes burnout might manifest in different patterns across individuals with T2D. We uncovered the distinct experiential dimensions of burnout in T2D—encompassing boredom, helplessness, anhedonia, as well as the transient joy derived from disengaging from prescribed diabetes care tasks—that emerge over the long term. The identified themes show clear convergence with several indicators in the Burnout Measure Short Version (20, 21) but are not adequately captured by established tools such as the DBS or Maslach Burnout Inventory (22).

Another key distinction of the DBS-T2D from existing diabetes burnout evaluation instruments (16, 20, 21, 24) lies in its emphasis on the stress experiences associated with diabetes management for T2D patients. This dimension was incorporated following a proposal by two general practitioners and two endocrinologists during the Delphi consultation process. Within this dimension, experts emphasized the importance of assessing a range of pressures faced by patients, including economic pressure, time pressure, and social pressure.

According to the IDF Diabetes Atlas 2025, the average healthcare expenditure per person with diabetes in China was estimated at $1, 141 for the year 2024 (1). Studies have shown that cost-related nonadherence is common among individuals with diabetes (36), particularly in relation to social determinants of health, including perceived financial stress, financial insecurity with health care (37), and adverse socioeconomic and health factors (38).

Regarding time pressure, the substantial investment of time, energy, and resources in diabetes self-management itself represents a heavy burden for patients. The Japanese Clinical Practice Guideline for Diabetes specifically recommends at least 150 minutes of physical activity per week for patients with T2D (39). Once the constant demands of daily self-management (e.g., specialized meal preparation, regular blood sugar checks, and insulin injections) are accounted for, the collective time burden imposes a far heavier load on patients than is often recognized. This burden directly leads to compromised adherence, resulting in simplified or abandoned self-care practices. This evidence robustly supports the pathway from time pressure, through diabetes distress and burnout, to decreased adherence and worsened clinical outcomes.

Furthermore, social pressure is also a critical component in the assessment of diabetes burnout. The relationship between social isolation and diabetes operates bidirectionally. Not only is social isolation a recognized risk factor for the onset of T2D (40), but the ongoing self-management and burden of complications can also lead to withdrawal from social activities (41, 42).

### Strengths and limitations

This study possesses several notable strengths. First, it represents the first assessment tool specifically designed to evaluate diabetes burnout in patients with T2D. Its items are grounded in the existing literature and tailored to the actual experiences and challenges of the T2D population, giving it greater ecological validity and clinical relevance compared to burnout scales borrowed from other fields. Second, the tool’s development was grounded in a robust, multi-stage methodological process. This included a systematic literature review, in-depth qualitative interviews with patients, and a structured Delphi expert consultation, ensuring comprehensive dimension coverage and establishing a solid foundation for content validity. Finally, one main objective of the Delphi process is to be inclusive of perspectives from a variety of stakeholders (43). The use of the Delphi method to construct the diabetes burnout scale in this study benefited from a diverse panel of experts, including endocrinologists, general practitioners from both comprehensive hospitals and community settings, and individuals living with T2D. This diversity ensured multi-faceted perspectives, greatly enhancing the robustness and practical relevance of the final indicator system.

This study also has several limitations. First, the preliminary literature review underpinning this study only included literature published in English and Chinese, potentially limiting the scope of global perspectives. Second, despite intentional efforts to achieve gender balance in participant recruitment, the majority of participating experts were female. This gender disparity is consistent with the higher proportion of female professionals in clinical medicine in China, particularly in the fields of internal medicine, gynecology, pediatrics, and general practice. Third, the geographical diversity of the respondents was limited. Most participants were based in Beijing, while representation from other provinces was relatively low, which may restrict the generalizability of the findings across different regions of China. Another limitation of the Delphi approach is that experts are limited to evaluating a predetermined list of items. Although the research team conducted a comprehensive preliminary phase—including a scoping review and semi-structured individual interviews—to identify potential items, the completeness of the initial list could not be fully ensured. To address this limitation, the survey included an open-ended section allowing experts to propose additional items, which contributed valuable insights (19). Finally, while the Delphi technique ensured the content validity of the scale, its reliability and construct validity have not yet been tested. To address this limitation, future research should focus on a comprehensive psychometric evaluation to confirm the measurement properties of this instrument in diverse T2D populations.

## Conclusion

This study successfully developed a diabetes burnout scale for patients with T2D using a modified Delphi method. The instrument aims to systematically identify and assess burnout experienced during diabetes self-management, providing a scientific basis for understanding and addressing this significant barrier to effective care. Future research will focus on validating the scale’s application in real-world clinical and community settings, while also exploring effective intervention strategies to mitigate diabetes burnout and improve patient experiences and health outcomes.

## Data Availability

The original contributions presented in the study are included in the article/supplementary material. Further inquiries can be directed to the corresponding author.
